# Iron Biofortification in Rice: An Update on Quantitative Trait Loci and Candidate Genes

**DOI:** 10.3389/fpls.2021.647341

**Published:** 2021-05-26

**Authors:** B. P. Mallikarjuna Swamy, Balram Marathi, Ana I. F. Ribeiro-Barros, Mark Ian C. Calayugan, Felipe Klein Ricachenevsky

**Affiliations:** ^1^International Rice Research Institute, Los Baños, Philippines; ^2^Agricultural College, Warangal, Professor Jayashankar Telangana State Agricultural University, Hyderabad, India; ^3^Forest Research Centre (CEF), Instituto Superior de Agronomia, Universidade de Lisboa, Lisbon, Portugal; ^4^Institute of Crop Science, University of the Philippines Los Baños, Laguna, Philippines; ^5^Departamento de Botânica, Instituto de Biociências, e Programa de Pós-Graduação em Biologia Celular e Molecular, Centro de Biotecnologia, Universidade Federal do Rio Grande do Sul, Porto Alegre, Brazil

**Keywords:** hidden hunger, biofortification, rice, grain, iron, QTLs, genes

## Abstract

Rice is the most versatile model for cereals and also an economically relevant food crop; as a result, it is the most suitable species for molecular characterization of Fe homeostasis and biofortification. Recently there have been significant efforts to dissect genes and quantitative trait loci (QTL) associated with Fe translocation into rice grains; such information is highly useful for Fe biofortification of cereals but very limited in other species, such as maize (*Zea mays*) and wheat (*Triticum aestivum*). Given rice’s centrality as a model for Poaceae species, we review the current knowledge on genes playing important roles in Fe transport, accumulation, and distribution in rice grains and QTLs that might explain the variability in Fe concentrations observed in different genotypes. More than 90 Fe QTLs have been identified over the 12 rice chromosomes. From these, 17 were recorded as stable, and 25 harbored Fe-related genes nearby or within the QTL. Among the candidate genes associated with Fe uptake, translocation, and loading into rice grains, we highlight the function of transporters from the YSL and ZIP families; transporters from metal-binding molecules, such as nicotianamine and deoxymugineic acid; vacuolar iron transporters; citrate efflux transporters; and others that were shown to play a role in steps leading to Fe delivery to seeds. Finally, we discuss the application of these QTLs and genes in genomics assisted breeding for fast-tracking Fe biofortification in rice and other cereals in the near future.

## Introduction

Hidden hunger affects more than two billion people worldwide and is among the major challenges to be addressed on a priority basis to achieve *Zero Hunger*, particularly in African, Asian, and Latin-American countries. Indeed, each year there are *ca.* three million deaths due to nutritional deficiencies, mainly proteins, vitamins, and minerals (FAO et al., 2019). Among the 22 essential trace elements, iron (Fe), zinc (Zn), selenium (Se), and iodine (I) deficiencies affect more than half of the world population (World Health Organization, 2002), the first being the most common nutritional disorder (Kennedy et al., 2003; IFPRI, 2015). Fe is crucial for the normal functioning of several biological processes in living organisms (White and Broadley, 2009; Abbaspour et al., 2014; Toxqui and Vaquero, 2015), mainly due to its major role in catalytic activities of many enzymes, such as those related to oxygen transport, electron transfer, oxi-reduction reactions, collagen biosynthesis, and vitamin D metabolism (Abbaspour et al., 2014; Toxqui and Vaquero, 2015).

According to the World Health Organization, the prevalence of anemia (insufficient number of red blood cells or oxygen-carrying capacity) ranges from 23% in developed countries to 52% in the developing world, and half of the cases are derived from Fe deficiency (World Health Organization, 2015). Although Fe deficiency anemia (IDA) affects all population groups, children and pregnant women are the most vulnerable targets (World Health Organization, 2008). Chronic IDA seriously compromises growth and development in children, impairing cognitive and motor development and enhancing susceptibility to infections (Gupta et al., 2016; Wieringa et al., 2016). In adults, anemia affects the immune system and causes fatigue and reduced physical and psychological performance (Failla, 2003; Camaschella, 2017). In any case, the extent of IDA in human health depends on a combined set of environmental, genetic, and physiological factors (Toxqui and Vaquero, 2015; Cappellini et al., 2019). Besides IDA, other types of anemia may be related to (i) active bleeding related to menstruation, wounding, gastrointestinal ulcers, and cancer; (ii) kidney disease related to the decrease in the hormone erythropoietin, involved in the production of red blood cells; (iii) obesity-related systemic inflammation that increases hepcidin, reducing Fe availability; (iv) alcoholism (premature destruction of defective red blood cells); and (v) sickle cell anemia and thalassemia, two genetically inherited diseases related to the abnormal production of hemoglobin (Abbaspour et al., 2014). Additionally, Fe deficiency is also a factor of risk for osteoporosis as it is a key component of enzymes involved in bone metabolism, i.e., biosynthesis of collagen, the main component of connective tissue.

In plants, Fe is also a crucial constituent of several proteins and enzymes involved in key pathways that sustain plant growth, development, and metabolism, and its deficiency is directly related to the decrease in crop performance (productivity and quality) (Lucena and Hernandez-Apaolaza, 2017; Tripathi et al., 2018). Among others, Fe nutrition has been related to plant tolerance to biotic and abiotic stresses (Aznar et al., 2015; Tripathi et al., 2018; Cesco et al., 2020), being a key element in photosynthesis (Balk and Schaedler, 2014; Krohling et al., 2016), which is affected by different stresses (Munns and Tester, 2008; Chaves et al., 2009). Additionally, due to its low solubility in soils, Fe availability to plants is quite low (Briat et al., 2015). Thus, with the current challenges of agriculture imposed by the current scenario of climate change, population growth, and undernourishment taken together, the development of strategies that allow the improvement of Fe concentration in crops is mandatory to achieve food and nutritional security.

Mineral supplements, food fortification, and crop biofortification are the three strategies that can be used to fight malnutrition. However, implementation of the first two strategies is complex and almost unaffordable in developing and less developed countries; the third strategy constitutes an effective and durable approach (Gómez-Galera et al., 2010; Murgia et al., 2012; Briat et al., 2015). Manipulation of the microbiome, such as using plant growth–promoting bacteria, holds promise to help improve plant nutrition, but it is still at the beginning regarding nutrient levels in edible seeds (Pii et al., 2016; Scagliola et al., 2021). Biofortification strategies include agronomic practices, breeding, and genetically engineered crops, separately or combined (Gómez-Galera et al., 2010). Although conventional breeding is a long-term strategy and transgenic approaches are controversial, costly, and time-consuming, marker assisted breeding (MAB) seems to be the most straightforward step to improve mineral quality of crops.

In this review, we summarize the current knowledge of quantitative trait loci (QTL) identified in multiple studies using different genotypes and review the known genes associated with Fe delivery and accumulation in rice grains. The combination of both is key to identifying the most likely genes to contribute to fast-track the development of Fe-biofortified crops.

## Genes Controlling Fe Translocation and Loading in Seeds

There is considerable interest in producing Fe-biofortified cereals for human consumption, and as a result, there are many research groups focused on understanding Fe homeostasis and mechanisms controlling Fe translocation and loading into seeds of cereal. Here, we briefly review the genes that are clearly shown to have a role in controlling Fe loading in seeds, whether directly or indirectly (Whitt et al., 2020). Other genes that have known functions and were used to generate transgenic, biofortified plants are not included (for a review, see Connorton and Balk, 2019).

Fe is translocated to seeds from two sources: directly from the soil solution through root uptake and remobilization from different tissues and organs during seed development (Sperotto, 2013; Che et al., 2019). Most of our knowledge about Fe homeostasis is focused on Fe transporters involved in root uptake/root radial movement and transcriptional regulators, whereas Fe homeostasis in shoots and seeds is less understood.

Plants from the Poaceae family use a chelation-based strategy for Fe acquisition, also called Strategy II. For that, phytosiderophores (PS—2′-deoximugineic acid, or DMA, is common in grasses) are secreted to the rhizosphere by major facilitator superfamily transporters (*OsTOM1*/*OsZIFL4* in rice; Nozoye et al., 2011; Ricachenevsky et al., 2011), which chelate Fe^3+^ and form Fe^3+^-PS complexes that are transported into root cells by Yellow Stripe-Like (YSL) family transporters (*OsYSL15* in rice; Inoue et al., 2009; Lee et al., 2009; Conte and Walker, 2012; Sperotto et al., 2012). Rice also uses Fe^2+^ transporters (*OsIRT1* and *OsIRT2*) in roots (Ishimaru et al., 2006). Non-Poaceae species employ a reduction-based strategy in which Fe^3+^ is reduced to Fe^2+^ by ferric-chelate reductase and enters into root cells by IRT/ZIP family transporters (Kobayashi and Nishizawa, 2012). Rice uses a combined strategy for Fe uptake, a trait that was recently shown to have evolved before the domestication of cultivated rice (Wairich et al., 2019). Evidence also points to other possible mixed mechanisms of Fe acquisition in eudicots (Xiong et al., 2013; Grillet and Schmidt, 2019).

The YSL gene family was first characterized in maize and found to be involved in acquisition of Fe^3+^-PS complexes from the soil (Curie et al., 2001). In rice, *OsYSL15* performs Fe^3+^-PS uptake into root outer cells, and its expression is increased under Fe deficiency in roots (Inoue et al., 2009; Lee et al., 2009). *OsYSL15* is also expressed in developing seeds (Inoue et al., 2009; Lee et al., 2009), and knockout *osysl15* plants show decreased Fe seed concentration although overexpression of *OsYSL15* results in the opposite phenotype (Lee et al., 2009). Although these data suggest that *OsYSL15* may be involved in the control of Fe concentration in seeds, it is not possible to separate the role of *OsYSL15* in Fe primary uptake and Fe loading based on the current evidence.

Nicotianamine (NA) is a ubiquitous metal-chelating non-proteinogenic amino acid in land plants. NA is a synthesized from three molecules of S-adenosyl-methionine by NA synthase (NAS) and can either be a substrate for phytosiderophore synthesis or chelate metals and function in long distance transport. In *A. thaliana*, four NAS genes were shown to have roles in Fe distribution, presumably through Fe-NA binding and translocation in the phloem (Klatte et al., 2009; see below about Fe-NA transporters). NAS genes from barley and rice were also overexpressed in soybean, tobacco, sweet potato, and rice (or expressed under control of endosperm-specific promotor) to increase Fe translocation to seeds with promising advances for biofortification (reviewed by Nozoye, 2018). Despite the usefulness of NAS genes in transgenic approaches, little is known about their specific physiological function in cereals.

Rice has three NAS genes, *OsNAS1*, *OsNAS2*, and *OsNAS3* (Inoue et al., 2003). From these, *OsNAS1* and *OsNAS2* are strongly upregulated under Fe deficiency, whereas *OsNAS3* is induced upon Fe excess (Nozoye, 2018). *OsNAS3* is shown to be important for Fe translocation within the plant because knockout *osnas3* plants have decreased Fe levels in flag leaves and seeds, whereas plants with increased expression of *OsNAS3* by activation tagging (*OsNAS3-D1*) show the opposite phenotype (Lee et al., 2009). The same knockout *osnas3* plants are shown to be more sensitive to Fe excess, and *OsNAS3-D1* plants are more tolerant to Fe deficiency (Lee et al., 2009; Nozoye et al., 2019), suggesting that endogenous NA is important for Fe translocation and detoxification under Fe toxicity conditions.

*OsYSL2* is an Fe^2+^-NA plasma membrane transporter that loads Fe into phloem cells (Koike et al., 2004) and is required for Fe translocation to seeds, especially to the endosperm (Ishimaru et al., 2010). Similarly, the plasma membrane-localized transporter *OsYSL13* is involved in Fe distribution from old leaves to younger leaves under Fe deficiency conditions. Loss-of-function plants for *OsYSL13* showed decreased Fe concentration in seeds, which indicates that long-distance transport of Fe is mediated by YSL family members and is important for controlling Fe concentration in seeds (Zhang et al., 2018b).

*OsYSL9* is shown to transport both Fe^2+^-NA and Fe^3+^-DMA and linked to the Fe deficiency response (Senoura et al., 2017). *OsYSL9* is strongly expressed in reproductive tissues, especially in the scutellum and inner regions of the endosperm during seed development. *OsYSL9*-knockdown plants show decreased Fe in embryos but increased Fe in the endosperm. Therefore, *OsYSL9* is involved in embryo Fe loading from the endosperm through the scutellum and might be a good target for biofortification (Senoura et al., 2017).

One of the most exciting findings in the quest to identify transporters that control Fe loading in cereal seeds is the functional characterization of vacuolar iron transporter (VIT) family genes in rice, *OsVIT1* and *OsVIT2* (Zhang et al., 2012). Both genes are homologous to *AtVIT1*, which controls Fe localization in *A. thaliana* (Kim et al., 2006). *OsVIT1* and *OsVIT2* were suggested to store Fe in vacuoles of flag leaf cells, decreasing Fe availability for translocation to developing seeds via phloem. This is consistent with the finding that *osvit1* and *osvit2* knockout mutant plants have increased Fe in seeds due to less Fe storage in leaf cell vacuoles and increased translocation (Zhang et al., 2012). This finding is confirmed in an independent work (Bashir et al., 2013). Moreover, both studies found that seeds of *osvit1* and *osvit2* changed Fe distribution within the embryo (Zhang et al., 2012; Bashir et al., 2013). Interestingly, *TaVIT2* (but not *TaVIT1*) overexpression using an endosperm-specific promoter increased Fe accumulation in the white flour fraction in wheat and barley grains, supporting the usefulness of these genes in biofortification by increasing endosperm sink strength (Connorton et al., 2017).

Regulation of Fe deficiency response is linked to the concentration of Fe in seeds. The *HRZ* [*Haemerythrin motif-containing Really Interesting New Gene (RING)- and Zinc-finger protein 1*] genes from rice are homologs of BRUTUS/BRUTUS-like proteins from *A. thaliana*, which are shown to be negative regulators of the Fe deficiency response at the post-transcriptional level (Hindt et al., 2017; Rodriguez-Celma et al., 2019). *OsHRZ1* and *OsHRZ2* knockdown plants showed tolerance to low Fe and grew better in calcareous soil, in which Fe is less available. Increased protein levels of Fe uptake transporters are a likely explanation for this phenotype because degradation of upstream transcription factors, presumably targets of *OsHRZ1* and *OsHRZ2*, is decreased (Kobayashi et al., 2013). Interestingly, the authors found that *OsHRZ1* and *OsHRZ2* knockdown plants show increased Fe concentration in brown rice, indicating that higher Fe uptake in roots can increase Fe loading in seeds indirectly.

The mitochondrial iron regulated (*MIR*) gene was found to indirectly control Fe concentration in rice seeds (Ishimaru et al., 2009; de Oliveira et al., 2020). Plants that have no functional *MIR* are unable to properly regulate Fe levels as they accumulate more Fe in roots, shoots, and seeds while having constitutively high expression of Fe uptake genes (Ishimaru et al., 2009). This is consistent with data showing that *OsIRT1* (the rice Fe^2+^ transporter involved in Fe uptake) overexpression leads to increased Fe in roots, shoots, and seeds (Lee and An, 2009). Therefore, increased Fe uptake by roots seems to indirectly affect Fe loading in seeds.

Another important step for Fe delivery to developing seeds is Fe redistribution from the node. The intricate vasculature of the node allows for nutrients, such as Fe, to be redirected from the xylem transpiration stream to panicles, which requires at least three intervascular transfer steps, and depends on transporters for many elements (Yamaji and Ma, 2014, 2017). *OsFRDL1* is a citrate transporter necessary for Fe transport to reproductive tissues. *OsFRDL1* is the functional equivalent of *A. thaliana AtFRD3*, i.e., citrate efflux to the xylem for Fe^3+^-citrate complex formation, a necessary step for Fe translocation from roots to shoots through the transpiration stream (Rogers and Guerinot, 2002; Green and Rogers, 2004; Yokosho et al., 2009; Roschzttardtz et al., 2011). *OsFRDL1* is also expressed in shoots, mainly in vascular tissues, including leaves, nodes, peduncle, rachis, filament of the anthers, and husk. Knockout plants for *OsFRDL1* show significantly decreased pollen viability and fertility compared with wild type. Interestingly, Fe deposition in the parenchyma cell bridges of the nodes, where Fe (and Zn) accumulates in wild type, is higher in mutant plants, whereas Fe concentration in flag leaves is lower. Taken together, the data suggest that *OsFRDL1* is important for Fe solubilization and transport to panicles from nodes (Yokosho et al., 2016).

Recently, *OsVMT*/*OsZIFL12* was linked to Fe (and Zn) translocation to grains (Che et al., 2019). *OsVMT* is localized in the vacuole and has DMA transport activity. At the vegetative stage, *OsVMT* is highly expressed in the exodermis and endodermis of roots, and at the reproductive stage, at the parenchyma cell bridges of the node I (Che et al., 2019). The authors suggest that *OsVMT* is involved in sequestering DMA into root vacuoles, which is necessary for Fe^3+^-DMA complex formation and subsequent export from vacuoles and loading in xylem, where Fe^3+^-DMA is translocated to shoots (Che et al., 2019). Accordingly, *osvmt* mutants show increased Fe and Zn concentration in polished seeds. The authors suggest that, because *OsVMT* is highly expressed in the parenchyma cell bridges, an anatomical region that accumulates Fe and Zn (Moore et al., 2014; Yamaji and Ma, 2019), the lack of functional *OsVMT* leads to higher DMA in the cytosol, which solubilizes more Fe (and Zn), increasing translocation and loading into seeds. The accumulation of DMA in polished seeds of mutant plants compared with wild-type (Che et al., 2019) supports this hypothesis. Therefore, rice uses both DMA and citrate to chelate Fe for translocation from nodes to developing seeds, suggesting that, as with other nutrients, control of nutrient transport in the node is key for Fe loading in seeds.

One important regulator of Fe, Zn, and protein levels in wheat (*T. turgidum* ssp. *durum*) seeds, a NAC transcription factor named *NAM-B1*, was described years ago. *NAM-B1* is non-functional in modern pasta wheat varieties, whereas in the ancestral wild emmer wheat (*T. turgidum* ssp. *dicoccoides*), it is fully functional. Introgression lines and RNAi experiments show that the reduced/loss of function of *NAM-B1* leads to delayed senescence and decreased Fe levels in grains, indicating that senescence timing is important for Fe translocation to seeds (Uauy et al., 2006). However, no gene with similar function was found in rice despite several efforts (Sperotto et al., 2009; Distelfeld et al., 2012; Jeong et al., 2013). Therefore, wild relatives of wheat are an interesting source of genetic variability for improving Fe concentration in cultivated wheat varieties, an approach that can be used with wild rice species (Ricachenevsky and Sperotto, 2016; Bierschenk et al., 2020; Wairich et al., 2020).

Although gene functional characterization has been prolific in the last few years, we are still lacking a comprehensive model of how rice plants transport Fe from root uptake to delivery to seeds. Major questions are still open, such as how many transporters are relevant for Fe (either Fe^3+^ or Fe^2+^) uptake in roots, how they work in concert to achieve optimal Fe nutrition, and how Fe is delivered and loaded in the developing seed. Moreover, there is no information on genes directly linked to natural variation in Fe seed concentration or to Fe homeostasis in general in rice. Increasing our basic gene function in Fe homeostasis, combined with the number of QTLs already identified, should help fill that gap in the future.

## QTLs Associated With Fe Concentration in Rice Grains

Increasing the bioavailable Fe concentration in the rice endosperm is the major goal of the rice Fe-biofortification program (Mayer et al., 2008; Shahzad et al., 2014). However, conventional breeding efforts to develop high-Fe rice have not been successful except for the release of a high-Fe rice variety NSIC Rc172 (MS13). This variety was developed by crossing IR72, a high-yielding rice variety, with a tall traditional rice land race, Zawa Bonday. It has higher levels of Fe in both brown and white rice and possesses excellent agronomic, grain, and cooking quality traits (Gregorio et al., 2000; Swamy et al., 2016). Even the bioefficacy feeding trials using this rice variety showed increased Fe status in the human body and made positive health impacts (Haas et al., 2005). But there were not many concerted efforts to scale up and disseminate this product, and it failed to upgrade the variety with improved tolerance to prevailing biotic and abiotic stresses.

The narrow genetic variation, complex genetic architecture, huge genotype and environmental interactions are the major constraints for developing a high-Fe rice by traditional breeding (Kawakami and Bhullar, 2018; Connorton and Balk, 2019; Ludwig and Slamet-Loedin, 2019). Therefore, understanding the molecular basis, particularly identification of causative genes linked to variation in Fe concentration in seeds of several rice genotypes is instrumental for generating biofortified cultivars. However, to date, no such gene was isolated despite the number of QTLs mapped. Here we summarize the known QTLs that could be useful for rice Fe biofortification (Table 1).

**TABLE 1:** List of QTLs and candidate genes reported for iron concentration in rice.

**Population**	**Parents**	**Lines**	**Env**	**Chr**	**No. of QTLs**	**QTLs**	***R*^2^ (%)**	**References**
Multiple crosses	–	–	–	7, 8, 9	3		19–30	Gregorio et al., 2000
DH	IR64/Azucena	129	1	2, 8, 12	3		14–18	Stangoulis et al., 2007
RILs	Zhenshan 97/Minghui 63	241	1	1, 9	2	*qFe-1, qFe-9*	11–26	Lu et al., 2008
ILs	Teqing/*O. rufipogon*	85	1	2, 9	2	***qFe2-1***, *qFe9-1*	5–7	Garcia-Oliveira et al., 2009
RILs	Bala/Azucena	105	1	1, 3, 4, 7	4	*qFe1, qFe3, qFe4, qFe7*	10–21	Norton et al., 2010
RILs	Madhukar/Swarna	168	1	1, 5, 7, 12	7	*qFe1.1, qFe1.2, qFe5.1, qFe7.1, qFe7.2, qFe12.1, qFe12.2*	69–71	Anuradha et al., 2012
DH	Chunjiang 06/TN1	120	2	1, 6, 8	3		11–22	Du et al., 2013
F_2_	Swarna/Madhukar	178	1	3, 4	3		1–13	Nagesh et al., 2013
RILs, ILs	Lemont/TeQing	280/123	2	1, 2, 3, 4, 5, 6, 7, 8, 10	13		3–5	Zhang et al., 2014
BILs	IR75862/Ce258; IR75862/Zhongguangxiang1	401	2	1, 2, 6, 7, 11	5	***qFe1***, *qFe2*, ***qFe6***, ***qFe7***, ***qFe11***	6–18	Xu et al., 2015
BILs	Xieqingzao × *O. rufipogon*	202	2	3, 6, 9	3	*qFe3, qFe6, qFe9*	6–28	Hu et al., 2016
BC_2_F_2_	Swarna × *O. nivara* (IRGC81832, IRGC81848)	245/227	1	1, 2, 3, 4, 6, 8, 11, 12	15	*qFe_1.__1_, qFe_1.__2_, qFe_1.__3_*, ***qFe_2.__1_***, *qFe_2.__2_*, ***qFe_3.__1_***, *qFe_3.__2_, qFe_4.__1_, qFe_6.__1_, qFe_8.__1_*, ***qFe_8.__2_***, *qFe_11_._1_, qFe_11_._2_, qFe_11_._3_, qFe_12_._1_*	4–25	Swamy et al., 2018b
Multiparent	MAGIC Plus	144	4	3, 7, 9, 10, 11	7	*qFe_3.__1_, qFe_3.__2_, qFe_7.__1_, qFe_9.__1_, qFe_9.__2_*, ***qFe_10_._1_***, *qFe_11_._1_*	9–14	Descalsota et al., 2018
DH	PSBRc82 × Joryeongbyeo; PSBRc82 × IR69428	130; 97	2	4	1	*qFe_4.__1_*	9	Swamy et al., 2018a
Panel	colored rice accessions	152	2	6, 12	2	*qFe_6.__1_, qFe_12_._1_*	10.3–10.6	Descalsota-Empleo et al., 2019a,b
BC_2_F_5_	RP Bio-226 × Sampada	111	2	1, 6	4	***qFe_1.__1_, qFe_1.__2_, qFe_6.__1_, qFe_6.__2_***	1–17	Dixit et al., 2019
RILs	PAU201 × Palman 579	177, 106	1	5, 7, 9	5	*qFE_5_._1_, qFE_5_._2_, qFE_5_._3_, qFE_7_._1_, qFE_9_._1_*	35–95	Kumar et al., 2019
DH	IR05F102 × IR69428	148	3	9, 12	2	*qFe_9.__1_, qFe_12_._1_*	12–13	Calayugan et al., 2020
DH	93-11 × Milyang 352	123	2	3	7	***qFe*_3–1_**, *qFe_3–2_*	11–17	Lee et al., 2020
DH	Goami 2’ × “Hwaseonchal”	110	1	1, 4, 6, 7, 11		*qFe_1.__1_, qFe_1.__2_, qFe_1.__3_, qFe4,1 qFe6, qFe7, qFe11*	12–41	Jeong et al., 2020

*DH, Double Haploids; RILs, Recombinant Inbred Lines; ILs, Introgression Lines; R2, Phenotypic Variance; Env, Environment. Bold denotes stable QTLs across different seasons, locations, environments, and populations.*

**FIGURE 1:**
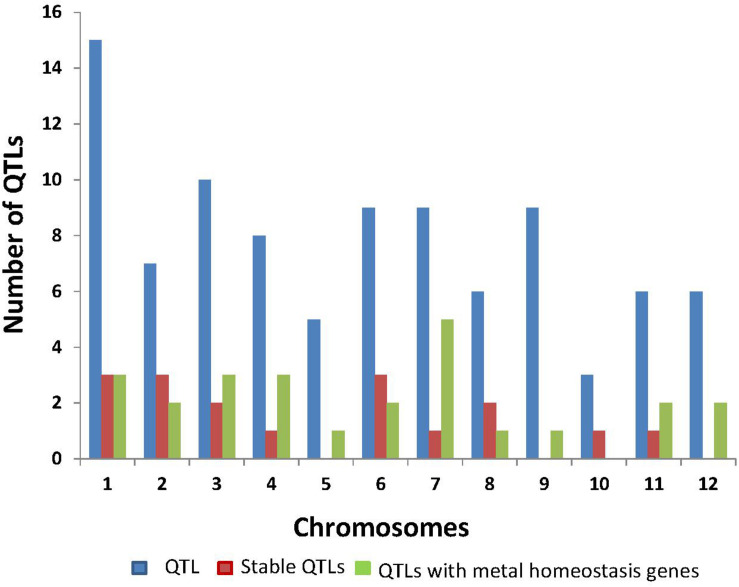
Summary of Fe QTLs and genes reported from different studies in rice.

We reviewed 20 published papers that focused on QTL identification for Fe concentration in rice and closely related species. Overall, 93 QTLs and 50 metal homeostasis-related candidate genes have been reported in rice with the highest number of QTLs reported on chromosomes 1, 3, and 7 (Figure 1 and Table 1). Seventeen Fe QTLs detected on chromosomes 1–4, 6–8, 10, and 11 were stable across different seasons, locations, environments, and populations. Some of the most prominent stable Fe QTLs are *qFe_1_, qFe_1.__1_, qFe_1.__2_, qFe_2–1_, qFe_3.__1_, qFe_3–1_, qFe_6.__1_, qFe_6.__2_, qFe_7_, qFe_8.__2_, qFe_10_._1_*, and *qFe_11_.* It is interesting to note that several positive QTL alleles for increased Fe concentration were contributed by wild rice species, such as *O. nivara* and *O. rufipogon*, deep water rice Madhukar and Jalamagna, and land races (Anuradha et al., 2012; Swamy et al., 2018b). The stability of the Fe QTLs in multiple populations and environments and their association with candidate genes involved in Fe homeostasis makes them useful for MAB or genomic selection (GS) and for further molecular characterization to understand the molecular mechanisms.

A total of 25 QTLs harbor known metal or Fe homeostasis-related candidate genes nearby or within the QTL. The highest number of reported Fe QTL with clear Fe homeostasis related candidate genes were on chromosomes 1, 3, 4, and 7 (Table 2). These genes were found to be involved in Fe acquisition in roots, biosynthesis of root exudates, uptake, translocation, and loading of Fe into different tissues/organs and to rice grains (Table 2). Some of the Fe homeostasis genes, such as *OsNAS1*, *OsNAS2*, *OsFer*, *OsVIT1*, *OsVIT2*, *OsZIP, OsIRO2*, and *OsIRT1*, have already successfully been cloned, and transgenics have been developed, which had up to a sixfold increase in Fe concentration and fourfold increase in Zn concentration compared to their respective base lines (Masuda et al., 2013; Kawakami and Bhullar, 2018). It is also interesting note that Fe and Zn loading pathways seem to be shared because increasing Fe usually results in increased Zn as well. Evidence for such shared pathways for Fe and Zn loading are emerging in other grasses, such as wheat (Astolfi et al., 2018). Still, most of the Fe accumulates in the brown rice, and a significant portion of the Zn accumulates in the endosperm (Johnson et al., 2011). Therefore, it might be feasible to generate biofortified rice varieties for both micronutrients simultaneously.

**TABLE 2:** Biological functions of QTL linked candidate genes for Fe concentration.

**QTL**	**Gene**	**Function**	**References**
*qFe_6.__1_*	*AtbZIP19, AtbZIP19*	Zinc accumulation in roots. Mediates the expression of the ZIP	Inaba et al., 2015
*qFe_12_._1_*	*APRT* (*Os12g0589100*)	Phosphate ion transmembrane transporter activity	Zhang et al., 2013
*qFe_4.__1_*	*OsFRO1* (*Os04g0578600*), *OsFRO2(LOC_Os04g48930*)	Fe absorption and homeostasis	Shou et al., 2019
*qFe_1.__2_*,	*OsZIP1* (*Os01g74110*)	Zinc ion transmembrane transport	Liu et al., 2019b
*qFe*_7_	*Os07g0510100, Os07g0517900, Os07g0518500, Os07g0519100, Os07g0519300, Os07g0519600, Os07g0521000, Os07g0529600, Os07g0556200*	Metal ion binding, Iron ion binding	https://www.uniprot.org/uniprot/Q6Z4B5
	*OsHMA7 (Os07g0623200)*	Copper-transporting ATPase	http://atgenie.org/gene?id=AT5G44790
*qFe_1.__2_, qFe_6.__1_*	*OsIAA5(LOC_Os01g48444.1)*	Auxin mediated signaling pathway	Liu et al., 2019a
*qFe_1.__2_, qFe_6.__1_*	*OsIAA6 (LOC_Os01g53880.1)*	Drought stress responses	Jung et al., 2015
*qFe_6.__1_*	*OsLCT1(LOC_Os06g38120.1)*	Involved in zinc and cadmium transport	Tian et al., 2019
*qFe_9.__1_*	*OsLysM-RLK10(LOC_Os09g33630.3) OsRLCK276*	ATP binding and protein self-association	https://www.uniprot.org
*qFe_5.__1_, qFe_3.__1_*	*OsMTP1 (Os05g0128400), OsMTP6 (Os03g0346800)*	Detoxification of zinc ion	https://www.uniprot.org/uniprot/A2XZZ6
*qFe_2.__1_*	*OsNAAT1 (Os02g0306401)*	Biosynthesis of mugineic acid	Inoue et al., 2008
*qFe_11_._1_*	*OsNAC5 (Os11g0184900)*	Transcription factors possibly controlling expression of metal-related genes	Sperotto et al., 2009
*qFe_3.__1_, qFe_7.__1_*	*OsNAS1 (Os03g0307300), OsNAS2 (Os03g0307200), OsNAS3 (Os07g0689600)*	Synthesizes nicotianamine, metal uptake, transport and loading	Inoue et al., 2003; Johnson et al., 2011; Lee et al., 2011; Trijatmiko et al., 2016
*qFe_1.__1_, qFe_7.__2_*	*OsNRAMP1 (Os07g0258400), OsNRAMP6 (Os01g0503400)*	Metal transporter controlling iron homeostasis	Curie et al., 2000; Peris-Peris et al., 2017
*qFe_4.__1_*	*OsOCP (LOC_Os04g55650.2)*	Metal uptake, transport, and loading	https://shigen.nig.ac.jp/rice/oryzabase/gene/detail/589.
*qFe_12_._1_*	*OsSWEET1, OsSWEET13*	Mediates both low-affinity uptake and efflux of sugar across the plasma membrane, haem binding	https://www.uniprot.org/uniprot/Q60EC2
*qFe_1.__2_, Fe_4.__1_, qFe_5.__1_, Fe_8.__2_*,	*OsYSL1 (LOC_Os01g13710.1), OsYSL4 (LOC_Os05g16290.1), OsYSL8(LOC_Os02g02460.1), OsYSL9 (LOC_Os04g45860.1), OsYSL16 (LOC_Os04g45900.1),OsYSL17 (LOC_Os08g17830.1)*	Transport of nicotianamine-chelated metals	Ishimaru et al., 2010; Sasaki et al., 2011; Inoue et al., 2009; Kakei et al., 2012
*qFe_1.__2_ Fe_5.__1,_ qFe_7.__2_,qFe_8.__2_*,	*OsZIP1* (*Os01g0972200*), *OsZIP4* (*Os08g0207500*), *OsZIP6* (*Os05g0164800*), *OsZIP7* (*Os05g0198400*), *OsZIP8* (Os07g0232800)	Zinc transporter that may mediate zinc uptake from the rhizosphere	Ramesh et al., 2003; Ishimaru et al., 2007; Ricachenevsky et al., 2018; Liu et al., 2019b

The narrow genetic variation for Fe concentration in polished rice in the readily useable primary gene pool of rice (i.e., species with AA genome, closely related to *Oryza sativa*) is a major constraint. There is a need to revisit the gene bank and screen gene bank accessions, especially wild rice and land races using more accurate and advanced Fe phenotyping protocols. This will help to identify potential high-Fe parental lines for discovery of major QTLs and use in breeding programs. Even though several major effect grain Fe QTLs explained very high phenotypic variance (>10%) and also gene-specific markers have been reported in rice (Table 1), there is no successful example of an Fe-biofortified rice genotype generated by QTL based MAB.

There is a huge potential to use these markers in MAB and GS to improve the grain Fe concentration in rice. Because there are multiple QTLs/genes responsible for grain Fe concentration located on different chromosomes, MAB through QTL pyramiding, rapid cycle recurrent selection (RCRS), and genomics assisted selection breeding approaches are worth trying to develop high-Fe rice. Genome wide association studies (GWAS) and GS approaches have not been explored much for improving grain micronutrients, but they hold great promise for improving the grain Fe concentration of several popular rice varieties and are highly useful in mainstreaming of rice Fe breeding. Moreover, genetic engineering and gene editing technologies have successfully demonstrated their potential to elevate levels of Fe in rice and improve bioavailability (Trijatmiko et al., 2016). Breeding programs have been initiated to transfer high-Fe traits using transgenic approaches into popular rice varieties through MAB (Paul et al., 2014; Moreno-Mayano et al., 2016).

Last, it is important to note that development of multiple nutrient-rich rice varieties with reduced levels of toxic elements, such as cadmium and arsenic, is also essential for the success of breeding for healthier rice (Van Der Straeten et al., 2020). Several advanced breeding materials, such as MAGIC populations, wild rice–derived introgression lines, and multicross-derived advanced breeding lines, are being developed, which are valuable genetic resources for genetic dissection of multiple nutrient elements (Swamy et al., 2016). Recently IRRI is leading the mainstream breeding for Zn biofortification, which aims to incorporate grain Zn as an important component of all future rice varieties. Similar efforts should be made for Fe mainstreaming in rice.

## Possible Candidate Genes With Known Function Within QTL Regions

Among the most promising candidate genes identified, we highlight transporters from the YSL gene family (Table 2). Besides the role of *OsYSL15* in Fe^3+^-PS transport (Inoue et al., 2009; Lee et al., 2009), other members were shown to perform Fe long-distance transport (Curie et al., 2009). *OsYSL9* has a role in Fe transport to rice grains, specifically in Fe transfer from the endosperm to the embryo because plants silenced for *OsYSL9* show decreased Fe concentrations in embryos but increased in other seed regions (Senoura et al., 2017). *OsYSL16* has been linked to transport of Cu-NA complexes (Zheng et al., 2012; Zhang et al., 2018a) and also play a role in Fe long-distance transport (Kakei et al., 2012). Recently, *OsYSL18* was shown to remobilize Fe from old to young leaves and to developing seeds (Zhang et al., 2018a). Therefore, the fact that some *OsYSL* genes coincide with QTLs is quite promising.

In agreement with a role of long-distance transport in determining final Fe concentration in seeds, there are QTLs colocalizing with the two *loci* that harbor the three NA synthase (NAS) genes (Table 2). Rice has three NAS genes (two of them are *in tandem*) for which the precise roles are not fully understood. Biofortification efforts using transgenics commonly increase NAS gene expression, presumably to increase NA-mediated Fe transport to developing seeds, a strategy that has been fruitful alone and in combination with other transgenes (Inoue et al., 2003; Johnson et al., 2011; Lee et al., 2011; Trijatmiko et al., 2016). It would not be surprising to find that NAS genes are linked to variation in Fe concentration in rice genotypes. One NA amino transferase (NAAT) gene (Inoue et al., 2008), which is involved in PS synthesis, was also found within a QTL (Table 2), highlighting how changes in metal chelators might be important for controlling Fe concentration in seeds.

The ZIP gene family also has promising candidate genes (Table 2). Among them, *OsZIP1* was suggested to detoxify Zn, Cd, and Cu from rice roots but not Fe (Liu et al., 2019b). *OsZIP4* was recently shown to function in Zn distribution to tiller buds and panicles (Mu et al., 2020). *OsZIP8* (Lee et al., 2010) and *OsZIP7* (Ricachenevsky et al., 2018; Tan et al., 2019; Gindri et al., 2020) both are Zn transporters with roles in Zn root-to-shoot translocation. However, none of these transporters is shown to be relevant for Fe homeostasis. *OsZIP6*, which also coincides with a QTL (Table 2), is shown to transport Fe (Kavitha et al., 2015) although its physiological role *in planta* is unknown. Still, given that Fe and Zn homeostasis do crosstalk and the translocation mechanisms to seeds seem to be at least partially shared, it is interesting to pursue whether these genes might explain variation in rice genotypes for Fe concentration in seeds.

Other interesting candidate genes are from the NRAMP gene family (Table 2). *OsNRAMP1* is part of the Fe deficiency regulon, being induced upon low Fe concentration in roots (Wairich et al., 2019). *OsNRAMP1* is very similar to *OsNRAMP5*, a well-known transporter for controlling Cd concentrations in seeds. *OsNRAMP1* was recently shown to transport Cd and Mn but not Fe or As as previously suggested (Chang et al., 2020). *OsNRAMP6*, on the other hand, is an Fe and Mn transporter, which undergoes alternative splicing. Both splicing isoforms can transport Fe and might be negatively linked to plant immunity (Peris-Peris et al., 2017).

Finally, we find that *OsHMA7* is within a QTL. The *OsHMA7* allelic variation was analyzed in recombinant inbred lines generated from crosses between Madhukar × Swarna, which show high and low Fe concentration in seeds, respectively (Kappara et al., 2018). Results show that lines silenced for *OsHMA7* or overexpressing either alleles result in complex phenotypes with changes in plant size and domestication traits. However, the over-expression of the allele from the high-Fe genotype results in increased Fe concentration in seeds, whereas the overexpression of the allele from the low-Fe genotype did not (Kappara et al., 2018). Therefore, *OsHMA7*, despite not having its molecular function characterized yet, is a good candidate gene for natural variation in Fe levels in rice grains.

## Conclusion

The QTLs and candidate genes reviewed here are a useful resource for future Fe biofortification efforts. From a practical standpoint, further QTL pyramiding using robust regions associated with high Fe concentrations might be feasible because many studies now support some of the same regions as linked to high Fe concentration in seeds. Still, efforts to identify causative genes and specific mutations linked to Fe accumulation in rice seeds would improve our understanding of the genetic basis for such variation, indicate which mechanisms are amenable to manipulation in rice plants for increasing Fe in seeds, and finally allow precise introgression of such genetic variants into elite genotypes using markers linked to causative mutations of the desired phenotypes. The information provided here will help future studies focused on such aims.

## Author Contributions

All authors contributed equally to writing the manuscript and reviewing and preparing the submission.

## Conflict of Interest

The authors declare that the research was conducted in the absence of any commercial or financial relationships that could be construed as a potential conflict of interest.
